# Metabolic Syndrome Parameters, Determinants, and Biomarkers in Adult Survivors of Childhood Cancer: Protocol for the Dutch Childhood Cancer Survivor Study on Metabolic Syndrome (Dutch LATER METS)

**DOI:** 10.2196/21256

**Published:** 2021-01-27

**Authors:** Vincent Pluimakers, Marta Fiocco, Jenneke van Atteveld, Monique Hobbelink, Dorine Bresters, Eline Van Dulmen-den Broeder, Margriet Van der Heiden-van der Loo, Geert O Janssens, Leontien Kremer, Jacqueline Loonen, Marloes Louwerens, Helena Van der Pal, Cécile Ronckers, Hanneke Van Santen, Birgitta Versluys, Andrica De Vries, Marry Van den Heuvel-Eibrink, Sebastian Neggers

**Affiliations:** 1 Princess Maxima Center for Pediatric Oncology Utrecht Netherlands; 2 Department of Internal Medicine Leiden University Medical Center Leiden Netherlands; 3 Mathematical Institute Leiden University Leiden Netherlands; 4 Department of Radiation Oncology University Medical Center Utrecht Utrecht Netherlands; 5 Department of Pediatric Oncology Emma Children’s Hospital/Amsterdam University Medical Center Amsterdam Netherlands; 6 Dutch Childhood Oncology Group Utrecht Netherlands; 7 Department of Hematology Radboud University Medical Center Nijmegen Netherlands; 8 Institute of Biostatistics and Registry Research Brandenburg Medical School Theodor Fontane Neuruppin Germany; 9 Department of Pediatric Oncology and Hematology Wilhelmina Children’s Hospital/University Medical Center Utrecht Utrecht Netherlands; 10 Department of Pediatric Oncology/Hematology Sophia Children’s Hospital/Erasmus Medical Center Rotterdam Netherlands; 11 Department of Endocrinology Erasmus Medical Center Rotterdam Netherlands

**Keywords:** metabolic syndrome, childhood cancer survivor, Dutch Childhood Cancer Survivor Study, methodology, Dutch LATER METS

## Abstract

**Background:**

Potential late effects of treatment for childhood cancer include adiposity, insulin resistance, dyslipidemia, and hypertension. These risk factors cluster together as metabolic syndrome and increase the risk for development of diabetes mellitus and cardio- and cerebrovascular disease. Knowledge on risk factors, timely diagnosis, and preventive strategies is of importance to prevent cardio- and cerebrovascular complications and improve quality of life. Currently, no national cohort studies on the prevalence and determinants of metabolic syndrome in childhood cancer survivors, including biomarkers and genetic predisposition, are available.

**Objective:**

The objectives of the Dutch LATER METS study are to assess 1) the prevalence and risk factors of metabolic syndrome and its separate components, and 2) the potential diagnostic and predictive value of additional biomarkers for surveillance of metabolic syndrome in the national cohort of adult long-term survivors of childhood cancer.

**Methods:**

This is a cross-sectional study based on recruitment of all survivors treated in the Netherlands between 1963 and 2002. Metabolic syndrome will be classified according to the definitions of the third Adult Treatment Panel Report of the National Cholesterol Education Program as well as the Joint Interim Statement and compared to reference data. Dual-energy x-ray absorptiometry scans were performed to assess body composition in more detail. The effect of patient characteristics, previous treatment, and genetic variation on the risk of metabolic syndrome will be assessed. The diagnostic and predictive value of novel biomarkers will be tested.

**Results:**

Patient accrual started in 2016 and lasted until April 2020. A total of 2380 survivors from 7 pediatric oncology hospitals have participated. From July 2020, biomarker testing, single nucleotide polymorphism analysis, and data analysis will be performed.

**Conclusions:**

The Dutch LATER METS study will provide knowledge on clinical and genetic determinants of metabolic syndrome and the diagnostic value of biomarkers in childhood cancer survivors. The results of this study will be used to optimize surveillance guidelines for metabolic syndrome in survivors based on enhanced risk stratification and screening strategies. This will improve diagnosis of metabolic syndrome and prevent complications.

**International Registered Report Identifier (IRRID):**

DERR1-10.2196/21256

## Introduction

Due to increasing survival of patients with childhood cancer, late side effects have become more prominent. Potential late effects include adiposity, insulin resistance, dyslipidemia, and hypertension, which cluster together as metabolic syndrome. Metabolic syndrome is associated with a higher risk of diabetes mellitus, as well as cardio- and cerebrovascular morbidity and mortality later in life [1-3]. The separate components are in themselves risk factors for diabetes and cardiovascular disease but, when coexisting, the components can aggravate each other, leading to an even higher risk of diabetes and cardiovascular disease [4,5].

Studies in childhood cancer survivors have reported a prevalence of metabolic syndrome of over 30% after 25 years follow-up, substantially higher compared to age- and sex-matched controls (odds ratio 1.76) [6,7]. This apparent risk difference for metabolic syndrome further increases the elevated risks for cardiovascular outcomes and endothelial damage from anthracyclines, alkylating agents, and irradiation [8,9]. Consequently, the mortality due to coronary and cerebrovascular disease in long-term survivors is up to 12.7 times higher than in the general population [10-13]. The fact that metabolic syndrome can be subclinical for many years emphasizes the need for timely identification of metabolic syndrome in survivors and early intervention strategies. Lifestyle and diet advice, exercise, and medication may prevent the development of diabetes and cardio- and cerebrovascular disease, improving survival rates and quality of life.

Several underlying conditions have been reported to increase the risk for (components of) metabolic syndrome in survivors: growth hormone deficiency, pancreatic beta cell dysfunction, hypogonadism, hypothyroidism, and altered body composition with increased intra-abdominal fat [14-19]. Hence, an increased risk of metabolic syndrome might be associated with treatment for a brain tumor, treatment with radiotherapy, intensive chemotherapy, nephrectomy, adrenalectomy, or stem cell transplantation [7,16,20-32]. The effects of other potentially harmful treatments, for example corticosteroids, and patient-related factors such as sex, age, body mass index at diagnosis, and lifestyle, are still not clear [3]. Also, heterogeneity in incidence of metabolic syndrome among homogeneously treated survivors suggests a role of genetic susceptibility [33,34]. A few studies using candidate gene approaches [24,35] as well as one genome-wide association study [36] have identified genetic variants that might be associated with development of metabolic syndrome and its components in survivors. Results based on these studies have not yet been replicated or functionally validated.

Multiple definitions of metabolic syndrome have been developed over the past years. The two most commonly used are those of the third Adult Treatment Panel Report of the National Cholesterol Education Program [37] and the Joint Interim Statement of the International Diabetes Federation; National Heart, Lung, and Blood Institute; and the American Heart Association [38]. Both definitions overlap largely but they differ in waist circumference cut-off point (Table 1). Apart from the 4 components, pro-inflammatory and prothrombotic markers have been reported to be relevant biomarkers of metabolic syndrome, as has hyperuricemia [39,40].

Adequate assessment of metabolic syndrome in survivors using the National Cholesterol Education Program and Joint Interim Statement definitions has specific challenges, particularly after abdominal radiotherapy. It has been shown that body mass index and waist circumference underestimate adiposity due to deformation of spine, muscles, and fat, particularly in past treatment eras when higher radiotherapy doses and larger fields were used [21,41,42]. Similarly, adiposity can be disguised due to sarcopenic obesity after stem cell transplantation [43,44]. Body composition can be more reliably measured by dual-energy x-ray absorptiometry, but this is time consuming and expensive to be implemented for standard follow-up of all survivors. Serum biomarkers may be more cost-effective surrogate markers for metabolic syndrome. In smaller survivor cohorts and in the general population, biomarkers other than triglycerides and high-density lipoprotein cholesterol that have been proposed as predictors of metabolic syndrome include low-density lipoprotein, apolipoprotein-B, leptin, adiponectin, uric acid, and C-reactive protein [39,45-50].

So far, large studies on clinically diagnosed metabolic syndrome in survivors are scarce. Two large multicenter cohort studies with clinically diagnosed metabolic syndrome are the American St. Jude Lifetime (SJLIFE, all types of childhood cancer) [6,7] and the French Leucémies de l'Enfant et l'Adolescent (leukemia) [31,51,52] studies. Other studies have yielded heterogeneous and sometimes conflicting results and can be difficult to compare. This may be due to metabolic syndrome components being analyzed only separately, or due to small patient cohorts, a questionnaire based or retrospective design, insufficient treatment data (eg, only childhood cancer diagnosis is known, not treatment), and short follow-up (metabolic syndrome risk increases continuously with age, so a follow-up of 10-20 years likely underestimates this) [22,32,53-55]. In addition, comparison of study outcomes can be difficult due to the use of different classifications. Currently, no studies in national cohorts on prevalence and determinants of metabolic syndrome in childhood cancer survivors, including biomarkers and genetic predisposition to metabolic syndrome, are available.

Here we describe the methodology of the Dutch LATER METS study in the adult cohort of survivors treated between 1963 and 2002. This nationwide study assesses metabolic syndrome prevalence, clinical and genetic risk factors, and the diagnostic and predictive value of additional biomarkers. The results of this study will be used to identify survivors at risk and to optimize surveillance guidelines.

**Table 1:** NCEP-ATP III^a^ and JIS^b^ classifications of metabolic syndrome, and alternative classification with adiposity measured by dual-energy x-ray absorptiometry scan.

Required for diagnosis (≥3)	Measurement	NCEP-ATP III	JIS	Alternative with dual-energy x-ray absorptiometry scan
Adiposity	Waist circumference (cm)	>102^c^/88^d^	≥94^c^/80^d^,^e^	Body fat Z-score >2
Insulin resistance	Fasting glucose (mmol/L)	≥5.5 or treatment	≥5.5 or treatment	≥5.5 or treatment
Dyslipidemia	Triglycerides (mmol/L)	≥1.7 or treatment	≥1.7 or treatment	≥1.7 or treatment
	High-density lipoprotein cholesterol (mmol/L)	<1.0^c^/1.3^d^ or treatment	<1.0^c^/1.3^d^ or treatment	<1.0^c^/1.3^d^ or treatment
Hypertension	Blood pressure (mmHg)	≥130/85 or treatment	≥130/85 or treatment	≥130/85 or treatment

^a^NCEP-ATP III = National Cholesterol Education Program Adult Treatment Panel III.

^b^JIS = Joint Interim Statement of International Diabetes Federation; National Heart, Lung, and Blood Institute; and the American Heart Association.

^c^Men.

^d^Women.

^e^Cut-off for Caucasian population.

## Methods

### Objectives

The objectives of this study are to assess 1) the prevalence and risk factors (patient characteristics, previous treatment, and genetic variation) of metabolic syndrome and its separate components, compared to reference data, and 2) the potential diagnostic and predictive value of novel biomarkers for surveillance of metabolic syndrome in the national cohort of adult long-term survivors of childhood cancer.

### Study Population and Design

**Figure 1:**
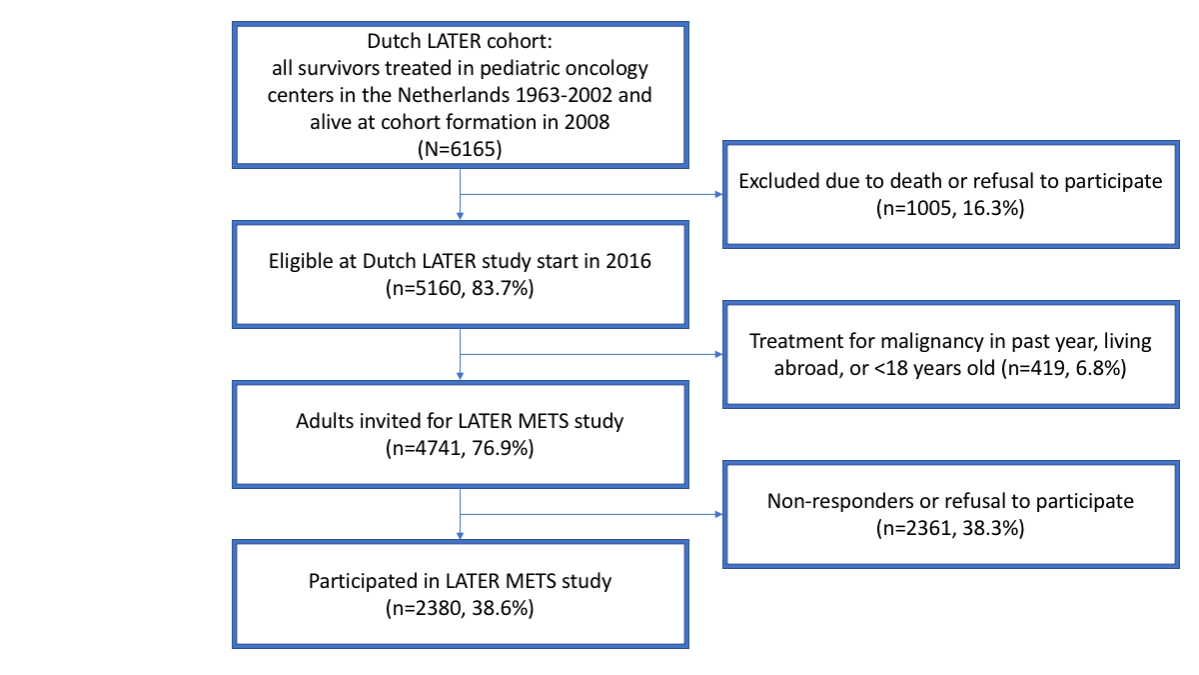
Overview of the Dutch LATER study cohort and embeddedness of the Dutch LATER METS study cohort within the underlying cohort. Percentages indicate proportion of Dutch LATER cohort (N=6165).

The Dutch LATER METS study is part of the nationwide Dutch LATER study (Figure 1). This study started accrual in all 7 pediatric oncology centers in the Netherlands in 2016, thereby inviting the national cohort of all survivors treated in these hospitals between 1963 and 2002 to participate. Survivors were identified from registries of children with newly diagnosed cancer that are maintained in each of the 7 pediatric oncology centers in the Netherlands. This study merged the available information to create a specific childhood cancer survivors registry containing all registered survivors. Dependent on completeness of the sources in the centers, the starting year varied from 1963 to 1977. The LATER METS study was approved by the Medical Research Ethics Committee of the Amsterdam University Medical Center (registered at toetsingonline.nl, NL32117.018.10).

In the Dutch LATER study, data from 15 substudies of late effects were collected, including cardiotoxicity, bone density, frailty, growth hormone deficiency, renal toxicity, fatigue, and psychological late effects. Individuals who survived at least 5 years after diagnosis of histologically confirmed malignancies (as defined in the 3rd edition of the International Classification of Childhood Cancer [56]) or Langerhans cell histiocytosis, were treated with chemotherapy or radiotherapy, and were between 0 and 17 years of age at diagnosis were invited. Exclusion criteria were treatment for a malignancy in the past year and living abroad.

For all eligible survivors, prior to the visit of the late-effects clinic, sex; date of birth; date of cancer diagnosis; and detailed data on cancer type and treatment, including chemotherapy regimens and doses, radiotherapy fields and (fractionated) dose, stem cell transplantation and corticosteroid treatments, were collected in a pseudonymized, web-based, central database. This includes primary diagnosis as well as, if present, recurrences and subsequent malignancies.

Subsequently, data collection for all studies was combined with the survivors’ regular care visit to the late-effects clinic for the majority of survivors. Before the visit, survivors received information about the study, sent by mail by the study personnel. If they agreed to participate, study data was collected by the treating physician or the study personnel.

The entire cohort, at formation in 2008, contained 6165 eligible survivors. By mail, survivors were provided the option to opt-out of future study participation. For the Dutch LATER study, the cohort was frozen in 2016, leaving 5160 subjects eligible. For the LATER METS study, only adults (n=4741) were invited. Inclusion took place until April 2020. Written informed consent was obtained from all study participants.

### Reference Population

Normative data from the Dutch Lifelines study cohort will serve as reference population [57]. This is a 3-generation cohort of 167,000 inhabitants (10%) of the north of the Netherlands, from whom, among other data, the following parameters relevant to our study were collected between 2006 and 2013: age, sex, height, weight, waist and hip circumference, blood pressure, comorbidities, medication use, smoking, physical activity, high-density lipoprotein, triglycerides, glucose, apolipoprotein-B, low-density lipoprotein, total cholesterol, uric acid, and high sensitivity C-reactive protein. We aim to use a subset of this reference cohort as controls that have the same age and sex distribution as our study cohort.

### Data Collection

#### Data Collected Before Visit of Late-Effects Clinic

An overview of collected variables is presented in Table 2. In addition to the previously mentioned data, the following variables relevant for the Dutch LATER METS study were extracted from the medical records: height and weight at cancer diagnosis and relevant comorbidities.

#### Data Collected at Visit of Late-Effects Clinic

Weight was measured without shoes and with light clothing on an electronic scale to the nearest 0.1kg. Height was measured without shoes to the nearest centimeter. Body mass index was calculated from weight and height. Waist circumference was measured in the middle between the lower rib and iliac crest to the nearest centimeter. Hip circumference was measured at the greater trochanter to the nearest centimeter. Waist/hip ratio was calculated. Blood pressure was measured after at least 5 minutes rest with an electronic oscillometric meter (the mean of two measurements).

Survivors completed a general health questionnaire, containing questions about comorbidities, current medication use, smoking and alcohol habits, education level, and family history of diabetes mellitus and cardiovascular disease. They also completed the Short Questionnaire to Assess Health enhancing physical activity [58]. Total body dual-energy x-ray absorptiometry scans (Hologic and Lunar types) were used to assess body composition [41]. These measurements include fat percentage and lean body mass. The 6-minutes walking test was performed in a subset of the survivors (those treated in the Sophia children’s hospital/Erasmus Medical Center, Rotterdam) as a measure of functional exercise capacity [59,60].

**Table 2:** Overview of collected variables.

Collection period	Category	Variable		Unit(s) or categories	
Collected before visit of late-effects clinic	Childhood cancer type and treatment	Primary childhood cancer diagnosis		ICCC-3^a^ classification	
		Treatment protocol		Name and arm	
		Chemotherapy, per regimen		TCD^b^	
		**Radiotherapy field**			
			Cranial/craniospinal		TCD, fractions (if applicable)
			Total body		TCD, fractions (if applicable)
			Abdominal		TCD, fractions (if applicable)
			Pancreas involvement		TCD, fractions (if applicable)
		**Surgery procedure**			
			Autologous stem cell transplantation		Yes / No, conditioning regimen
			Allogeneic stem cell transplantation		Yes / No, conditioning regimen
		Relapse		Yes / No	
	Patient characteristics	Sex		Male / Female	
		Date of birth		Date	
		Date of childhood cancer diagnosis		Date	
		Date of study measurements (follow-up date)		Date	
	Medical history	Height at cancer diagnosis		Centimeter	
		Weight at cancer diagnosis		Kilogram	
		Growth hormone deficiency		Yes / No	
		Growth hormone replacement		Yes / No	
		Hypothyroidism		Yes / No	
		Hypogonadism		Yes / No	
		Hypocortisolism with steroid replacement		Yes / No	
Collected at visit of late-effects clinic	Physical examination	Height		Centimeter	
		Weight		Kilogram	
		Waist circumference		Centimeter	
		Hip circumference		Centimeter	
		Blood pressure		mmHg	
	General health questionnaire	**Does the survivor have or has the survivor experienced**			
			High cholesterol		Yes / No, age at diagnosis
			Hypertension		Yes / No, age at diagnosis
			Diabetes mellitus		Yes / No, age at diagnosis
			Myocardial infarction		Yes / No, age at diagnosis
			Stroke		Yes / No, age at diagnosis
		Medication use		Type, dose, age at start	
		Smoking status		Yes / Former / No	
		Cardiovascular disease in family		Relative, type of disease, age at diagnosis	
	Questionnaire to Assess Health enhancing physical activity	N/A^c^		N/A	
	Dual-energy x-ray absorptiometry scan	Total body fat		Percentage	
		Z-score total body fat		Z-score	
		Lean body mass		Kilogram per m^2^	
		Appendicular lean body mass		Kilogram per m^2^	
	6-minute walking test	N/A		Meter	
	Data determined from stored samples	**Serum biomarkers**			
			High-density lipoprotein		mmol/L
			Low-density lipoprotein		mmol/L
			Total cholesterol		mmol/L
			Apolipoprotein-B		g/L
			Glucose		mmol/L
			Insulin		pmol/L
			Adiponectin		ug/mL
			Leptin		ng/mL
			Uric acid		mmol/L
			High sensitivity C-reactive protein		mg/L
			IL-6^d^		pg/mL
			hsTNFa^e^		pg/mL
			IL-1^f^		pg/mL
			IGF-1^g^		ug/L
			Creatinine		mg/mmol
			Urea		mmol/L
			LH^h^		U/L
			FSH^i^		U/L
			AMH^j^		ug/L
			Estradiol		pmol/L
			Testosterone		nmol/L
		DNA from blood/saliva		N/A	

^a^ICCC-3: International Classification of Childhood Cancer, edition 3.

^b^TCD: total cumulative dose.

^c^N/A: not applicable.

^d^IL-6: interleukin-6.

^e^hsTNF: high-sensitivity tumor necrosis factor alpha.

^f^IL-1: interleukin-1.

^g^IGF-1: insulin-like growth factor 1.

^h^LH: luteinizing hormone.

^i^FSH: follicle stimulating hormone.

^j^AMH: anti-Müllerian hormone.

#### Data Determined From Stored Samples

Venous blood samples were drawn after overnight fasting and stored at -80°C in the biobank. To assess dyslipidemia, a lipid spectrum will be measured, consisting of triglycerides, high-density lipoprotein, low-density lipoprotein, total cholesterol, and apolipoprotein-B. Insulin resistance will be assessed by measuring glucose and insulin. Additionally, adiponectin, leptin, and uric acid will be measured. Inflammatory markers include high sensitivity C-reactive protein, interleukin-6, high sensitivity tumor necrosis factor alpha, and interleukin-1. The following possible confounders will be measured: insulin-like growth factor 1, kidney function (creatinine, urea), sex hormones (luteinizing hormone, follicle stimulating hormone, anti-Müllerian hormone in women, estradiol in women, testosterone in men), thyroid function (thyroid-stimulating hormone, free thyroxine), cortisol.

DNA for analysis of single nucleotide polymorphisms will be isolated from blood or, in survivors who received allogeneic stem cell transplantation, saliva. Saliva was obtained by spitting into a collection tube (Oragene kit) after not drinking or eating for 30 minutes.

### Metabolic Syndrome Definition

Metabolic syndrome will be classified according to definitions by the third Adult Treatment Panel Report of the National Cholesterol Education Program [37] and the Joint Interim Statement of the International Diabetes Federation; National Heart, Lung, and Blood Institute; and the American Heart Association [38] (Table 1). Should these criteria be updated during our analysis, we will strive to take these adjustments into account.

### Risk of Bias

Sex, date of birth, date of cancer diagnosis, and disease and treatment data are also available for nonparticipating survivors. Hence, comparing participating and nonparticipating survivors in order to determine the risk of selection bias is feasible. We will also compare these data between survivors with complete and incomplete data to judge the risk of attrition bias. Neither physician nor study personnel were blinded to the exposures of the survivors. Objectively measurable outcomes will reduce the risk of bias in this setting.

### Statistical Analysis

#### Prevalence of Metabolic Syndrome

The percentage of subjects with metabolic syndrome and the separate components will be assessed in survivors and in the Lifelines reference cohort according to both aforementioned metabolic syndrome definitions. Both cohorts will be compared by Chi-square (or Fisher exact) test. The relative risk for survivors to develop metabolic syndrome, compared to Lifelines reference data, will be calculated by employing a log-binomial regression model. The agreement between both metabolic syndrome definitions will be investigated with *kappa* statistic, in the whole cohort and stratified by sex.

A total body fat percentage of more than two standard deviations above the mean, as assessed by dual-energy x-ray absorptiometry, will be used as the most reliable marker for adiposity. We will estimate the correlation between waist circumference and fat percentage measured by dual-energy x-ray absorptiometry scan, and we will compare overweight classification with both definitions.

#### Risk Factors

Treatment-related risk factors for occurrence of metabolic syndrome and the separate components will be assessed using multiple uni- and multivariable logistic regression models. Based on literature, an initial model will be built with cranial radiotherapy, abdominal radiotherapy, and alkylating agents (total alkylating dose calculated using cyclophosphamide equivalent dose [61]) as treatment-related independent variables, and age, sex, follow-up time, and smoking as patient-related independent variables. The effect of potential additional risk factors will be assessed by adding them to the initial model, and variables with a *P* value <.20 will be kept in the final model. These potential risk factors include all other chemotherapy agents (type and total cumulative dose), other radiotherapy fields (body location and dose), corticosteroids, education level, family history, physical activity, functional exercise capacity, and comorbidities.

We will also investigate different abdominal radiotherapy fields involved (pancreas, liver), and the influence of stem cell transplantation conditioning regimens. We will also study patient- and treatment-related risk factors for the outcome underdiagnosis of overweight measured by waist circumference.

#### Biomarkers

Biomarker values will be reported, with reference values from the local laboratory where the samples are measured. This will be compared to Lifelines reference data by Chi-square (or Fisher exact) test. A risk factor analysis of altered biomarker values will be performed similarly to the abovementioned strategy for risk factor analysis of metabolic syndrome occurrence.

The diagnostic and predictive value of the biomarkers to detect metabolic syndrome will be investigated in multiple steps. We will stratify the survivors by metabolic syndrome presence or absence and compare mean or median values with the *t* test or Mann-Whitney U test. We will evaluate sensitivity and specificity and positive and negative predictive value based on the reference values of the local laboratory where the samples are measured. We will compare the area under the curve for a model with metabolic syndrome components and for a model with each biomarker added in order to investigate the additional diagnostic value of the novel biomarkers. We will build multivariable logistic regression models with metabolic syndrome as dependent variable and the biomarker as independent variable. In these models, we will also include metabolic syndrome components as covariates in order to investigate the independent predictive value of the novel biomarkers. We will estimate the metabolic syndrome risk by including the biomarker as categorical as well as continuous variable.

Correlation (Pearson or Spearman) between biomarkers and fat percentage by dual-energy x-ray absorptiometry scan will be used to measure the potential use as surrogate markers for adiposity.

#### Genetic Susceptibility Analysis

Genotyping will be performed with the Infinium Global Screening Array [62] on DNA isolated from blood or, in post-stem cell transplantation survivors, saliva. Quality control of the genotype data will be performed following a standardized protocol [63] including filtering based on call rate (excluded when <0.975 for either single nucleotide polymorphism or individual call rate), Hardy-Weinberg equilibrium, excess heterozygosity, gender mismatches, and familial relationships. Genetic ancestry will be assessed based on principal component analysis. Imputation will be performed with the Michigan Imputation Server using standard settings [64] with reference panel Haplotype Reference Consortium version r1.1 [65].

The single nucleotide polymorphism analysis will be performed with the RVtests software package [66] using multiple logistic regression models with metabolic syndrome and its separate components as outcomes. The initial analysis will be adjusted for age at follow-up, sex, and genetic ancestry. Then, potentially relevant covariates will be added to the model using forward selection to study whether they influence the single nucleotide polymorphism analysis; if so, they will be kept in the model. These covariates include: body mass index at follow-up, comorbidities (growth hormone deficiency, hypogonadism, diabetes mellitus, and hypothyroidism), cranial and abdominal radiotherapy, and alkylating agents (cyclophosphamide equivalent dose). We will also perform a time-to-event analysis (with left-censoring) on identified hits in order to get clinically relevant effect estimates.

Quality control of the single nucleotide polymorphism analysis will be performed with the EasyQC package using standard settings [67]. This includes filtering based on minor allele frequency (excluded when <0.05) and imputation quality (excluded when <0.3).

Visualization of the genetic associations and annotation of biological function for the top single nucleotide polymorphisms will be performed with the FUMA platform [68]. Findings will be replicated in available independent international cohorts.

## Results

### Patient Accrual

Patient accrual started in 2016 and lasted until April 2020. A total of 2380/4741 survivors have participated (participation rate 50.2%). From July 2020, biomarker testing, single nucleotide polymorphism analysis, and data analysis will be performed.

### Power Calculation

We performed a power calculation with an expected prevalence of metabolic syndrome in our study cohort of 30%. This percentage is based on results from the SJLIFE cohort, in which the prevalence of clinically diagnosed metabolic syndrome in 1598 survivors, after a mean of 25.6 years since diagnosis, was 31.8% [6]. This is the only large cohort study so far with clinically diagnosed metabolic syndrome in survivors of heterogeneous malignancies with a follow-up time comparable to that of our cohort.

Based on the sample size of 2380 survivors, expected metabolic syndrome prevalence of 30%, power of 80%, and type I error of .05, we will have sufficient power to detect an approximate 3% difference in metabolic syndrome prevalence with the reference cohort. For risk factor analysis among survivors, the minimum detectable difference will depend on in how many survivors the risk factor (eg, treatment regimen) is present. For example, if the risk factor is present in 10%, 25%, or 50% of survivors, a minimum difference of approximately 9%, 7%, or 6%, respectively, can be detected.

A genetic power calculator was used to estimate the relative risk that can be found in the genetic susceptibility analysis for an assumed minor allele frequency of 0.25 [69]. Based on the sample size of 2380, metabolic syndrome population prevalence of 15% [70], a power of 80%, a type I error of 5X10^–8^, and a case-control ratio of 1:2, the relative risk per high risk allele that can be found is 1.5.

## Discussion

In the current study, we will assess the prevalence and patient- and treatment-related risk factors for metabolic syndrome and its separate components in adult survivors of childhood cancer, as well as the additional diagnostic value of novel biomarkers for surveillance, and the genetic susceptibility to (treatment-related) metabolic syndrome by single nucleotide polymorphism analysis.

A total of 2380 survivors have participated in the study. This corresponds to 38.6% of all survivors (N=6165) in the Dutch LATER cohort, and a participation rate of 50.2% of invited adult survivors (n=4741). The definitive numbers of refusals, nonresponders, deaths or otherwise excluded subjects are not available yet. We will report these in the paper with the results of our study.

Strengths of this study include the availability of a national cohort of survivors, the availability of comprehensive disease and treatment data, and the clinical assessment of late effects, in addition to questionnaire based endpoints. So far, the role of biomarkers and genetic susceptibility to metabolic syndrome has not been well defined in survivors. We specifically intend to use dual-energy x-ray absorptiometry scans and relevant biomarkers (those with a high independent diagnostic or prognostic value, and a high correlation with fat percentage on dual-energy x-ray absorptiometry scan) to enable identification of survivors at risk for metabolic syndrome, in whom waist circumference measurement is not feasible due to abdominal radiotherapy.

In conclusion, our study will provide knowledge on clinical and genetic determinants of metabolic syndrome and the diagnostic value of biomarkers in adult childhood cancer survivors. The results of this study will be used to optimize surveillance guidelines for metabolic syndrome among survivors, based on enhanced risk stratification and screening strategies. This will improve the diagnosis of metabolic syndrome and prevent complications, thereby improving quality of life.
